# Vagus nerve stimulation attenuates acute kidney injury induced by hepatic ischemia/reperfusion injury in rats

**DOI:** 10.1038/s41598-022-26231-w

**Published:** 2022-12-15

**Authors:** Simin Deng, Yifeng Zhang, Ying Xin, Xinqun Hu

**Affiliations:** Department of Cardiovascular Medicine, The Second Xiangya Hospital, Central South University, 139 Middle Renmin Road, Changsha, 410011 Hunan China

**Keywords:** Medical research, Pathogenesis

## Abstract

Hepatic ischemia/reperfusion (I/R) injury, caused by limited blood supply and subsequent blood supply, is a causative factor resulting in morbidity and mortality during liver transplantation and liver resection. Hepatic I/R injury frequently contributes to remote organ injury, such as kidney, lung, and heart. It has been demonstrated that vagus nerve stimulation (VNS) is effective in remote organ injury after I/R injury. Here, our aim is to investigate the potential action of VNS on hepatic I/R injury-induced acute kidney injury (AKI) and explore its underlying mechanisms. To test this hypothesis, male Sprague–Dawley rats were randomly assigned into three experimental groups: Sham group (sham operation, n = 6); I/R group (hepatic I/R with sham VNS, n = 6); and VNS group (hepatic I/R with VNS, n = 6). VNS was performed during the entire hepatic I/R process. Our results showed that throughout the hepatic I/R process, VNS significantly regulated the expression levels of various iconic factors and greatly enhanced the protein expression levels of nuclear factor erythroid 2-related factor 2 (Nrf2) and hemeoxygenase-1 (HO-1) in the kidneys. These findings suggested that VNS may ameliorate hepatic I/R injury-induced AKI by suppressing inflammation, oxidative stress, and apoptosis probably through activating the Nrf2/HO-1 signaling pathway.

## Introduction

As a two-stage pathophysiological process, hepatic ischemia/reperfusion (I/R) injury is a leading cause of acute liver failure and is a severe clinical complication that inevitably occurs in complex liver surgeries, such as liver transplantation (LT) and major liver resection^1^. In addition to acute liver injury, hepatic I/R injury often causes remote organ injury, particularly in acute kidney injury (AKI)^2^. AKI is frequently associated with increased mortality, prolonged hospital stays, and higher healthcare costs in patients suffering from LT^3,4^. In addition, during the peritransplant period of LT, 78% of patients with AKI have a five-fold increased risk of death^5^.

The etiology of AKI is complex and multifactorial, mainly attributed to acute ischemia and acute hypoxia caused by hemodynamic instability^6^. However, the pathomechanisms remain incompletely characterized. Although many advances have been made in the management of hepatic I/R injury over past decades, the morbidity and mortality rates associated with hepatic I/R injury-induced AKI remain high and are of increasing concern. Hepatic I/R injury accompanied with AKI is an independent prognostic factor of AKI around a 40% rate, depending on the diagnosis and heterogeneity^7^. Few specific preventive and therapeutic options exist and there is an urgent need to seek novel effective approaches to reduce the incidence and severity of hepatic I/R injury-induced AKI.

Vagus nerve stimulation (VNS), as an FDA-approved therapeutic measure, is a well-tolerated adjunctive treatment for medically refractory partial-onset epilepsy and treatment-resistant depression^8^. Since 1989, the year of the first human VNS implantation, more than 100,000 patients have been treated with VNS worldwide. Additionally, VNS has been proven to reduce I/R injury in multiple organs, including the kidneys, brain, and heart^9–11^. Furthermore, an increasing number of researchers are becoming aware of the potential of VNS on remote organ injury after I/R injury^12–14^. However, to the best of our knowledge, whether VNS can also reduce remote kidney injury after hepatic I/R injury remains unknown. The current study aimed to investigate whether VNS can protect against AKI induced by hepatic I/R injury and to explore its potential mechanisms.

## Results

### VNS significantly protected against AKI after hepatic I/R injury

**Figure 1:**
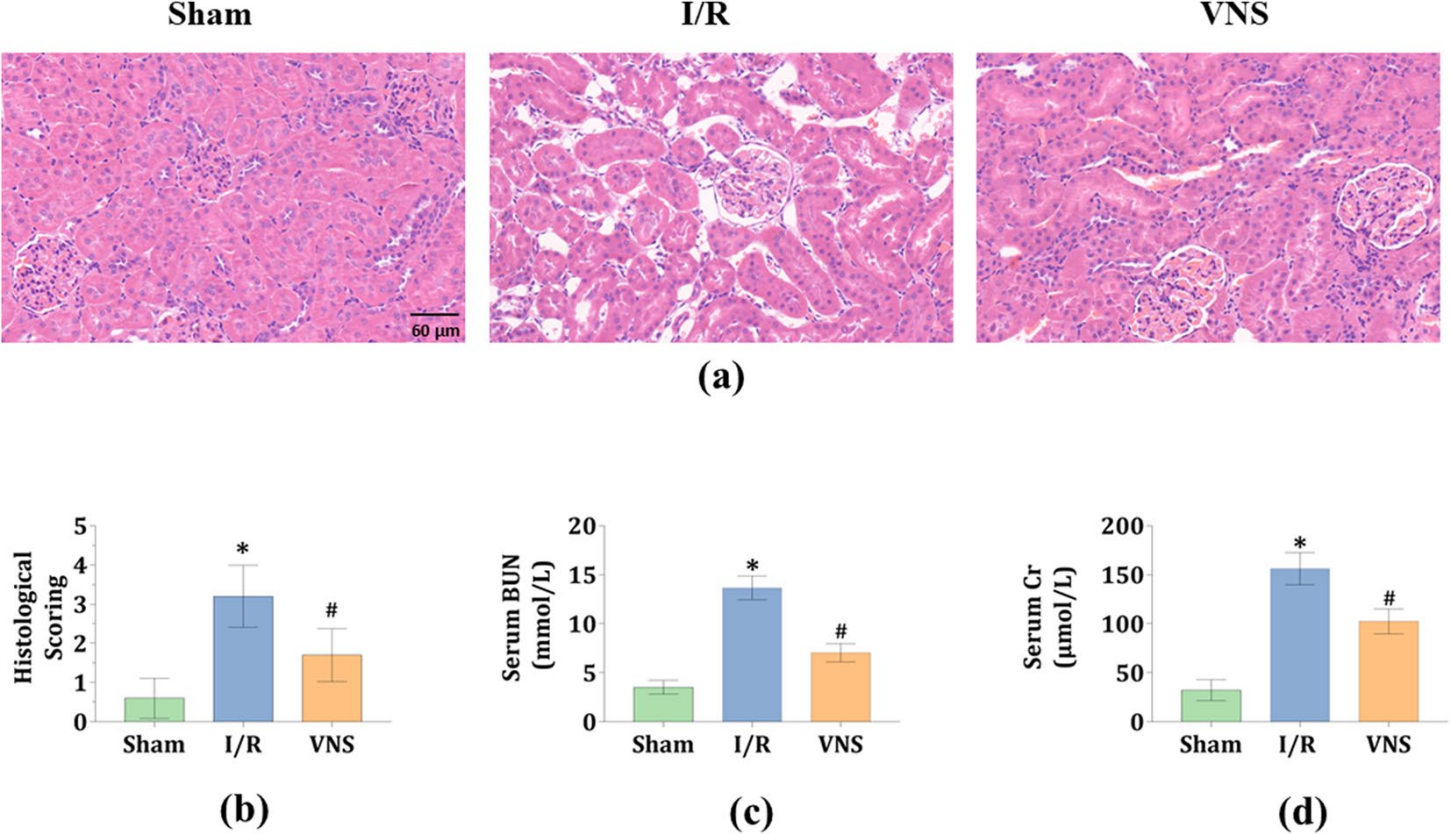
VNS alleviates kidney injury. (**a**) Representative H&E staining images of kidney tissues in the three groups are shown. (**b**) Kidney histological scores are shown. Levels of serum (**c**) BUN and (**d**) Cr are presented. *p < 0.05 vs. the Sham group; ^#^p < 0.05 vs. the I/R group. *H&E* hematoxylin and eosin, *BUN* Blood urea nitrogen, *Cr* creatinine. Light microscope (× 400 magnification).

Kidney injury was determined via histological examination and blood detection. Upon histological examination, there were no detectable alterations in the kidneys of the Sham group (see Fig. 1a). There were several hallmark signs of kidney damage in the I/R group, including tubular necrosis, loss of brush borders, cast formation, and tubular dilatation (see Fig. 1a). In contrast, compared to the I/R group, partial recovery was observed in the tubular cells of the VNS group (see Fig. 1a). Parallelly, compared to the Sham group, higher histological scores were observed in the I/R group (see Fig. 1b). However, lower histological scores were observed in the VNS group than in the I/R group (see Fig. 1b). Consistent with the histological changes, the serum blood urea nitrogen (BUN) and creatinine (Cr) levels were markedly higher in the I/R group than in the Sham group (see Fig. 1c,d). When compared with the I/R group, the VNS group exhibited a significant reduction in serum BUN and Cr levels (see Fig. 1c,d). These data indicate that VNS may exert a protective effect on the kidneys after hepatic I/R injury.

### VNS Significantly inhibited inflammation in the serum and kidneys after hepatic I/R injury

**Figure 2:**
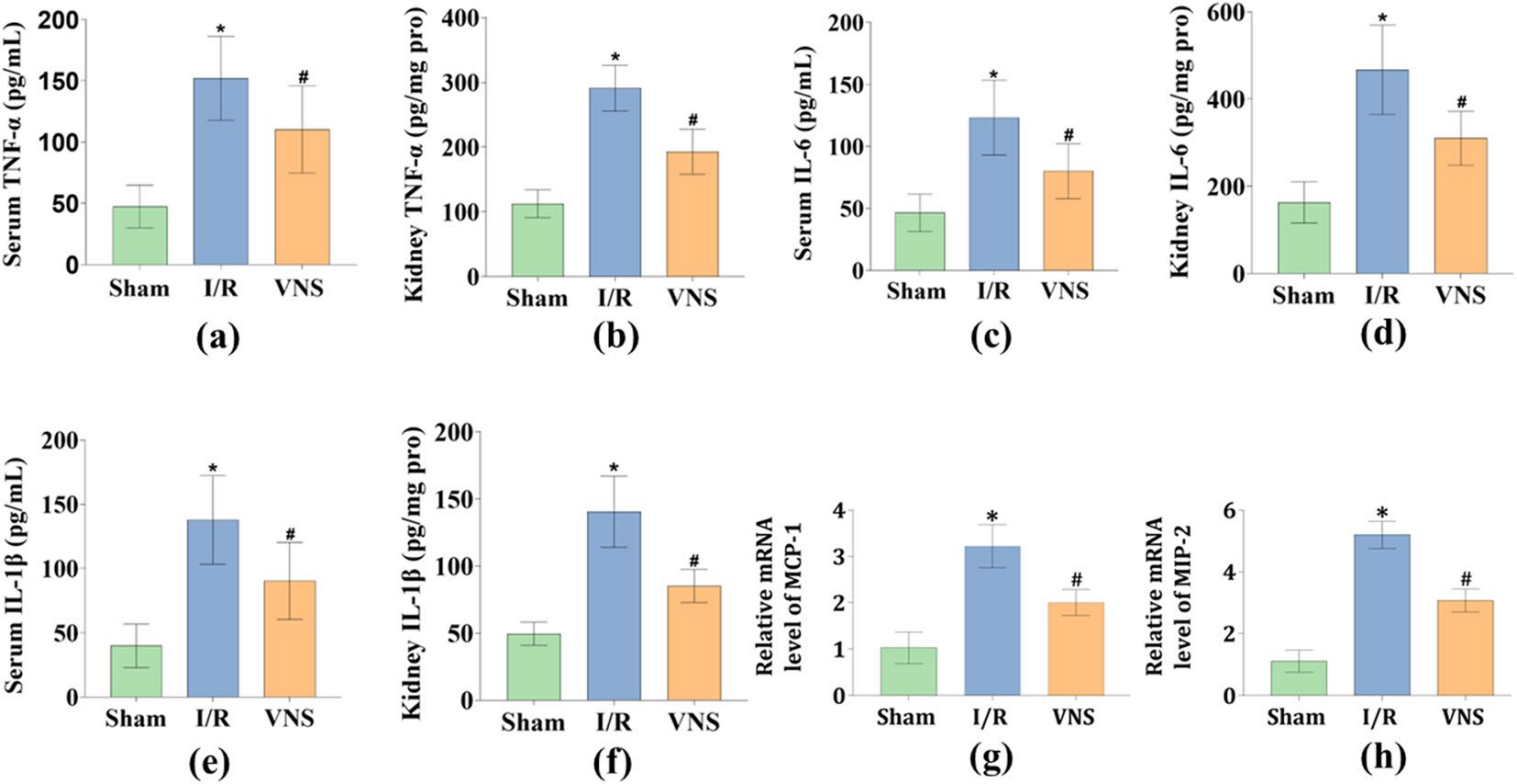
VNS mitigates inflammation in the serum and kidneys after hepatic I/R injury. The effect of VNS on the serum and kidney tissues levels of (**a**, **b**) TNF-α, (**c**, **d**) IL-6, and (**e**, **f**) IL-1β is shown. The relative mRNA levels of (**g**) MCP-1 and (**h**) MIP-2 in kidney tissues from the three groups are shown. Data are presented as mean ± SD. *p < 0.05 versus the Sham group; ^#^p < 0.05 versus the I/R group. *TNF-α* tumor necrosis factor alpha, *IL-6* interleukin 6, *IL-1β* interleukin1-beta, *MCP-1* monocyte chemoattractive protein 1, *MIP-2* macrophage inflammatory protein 2.

The levels of inflammatory cytokines in the kidneys were measured to examine whether VNS could modulate inflammation in the kidneys after hepatic I/R injury. The kidney tissues levels of tumor necrosis factor alpha (TNF-α), interleukin 6 (IL-6), and interleukin1-beta (IL-1β) were significantly increased in the I/R group compared to those in the Sham group (see Fig. 2b,d,f). In contrast, when compared with the I/R group, VNS treatment greatly lowered the kidney tissues levels of TNF-α, IL-6, and IL-1β (see Fig. 2b,d,f). In addition, we measured the serum levels of inflammatory cytokines to examine whether VNS could modulate systemic inflammation after hepatic I/R injury. And the serum levels of TNF-α, IL-6, and IL-1β presented the same variation trend as kidney tissues (see Fig. 2a,c,e).

Monocyte chemoattractive protein 1 (MCP-1) and macrophage inflammatory protein 2 (MIP-2) mRNA levels were evaluated by quantitative real-time polymerase chain reaction (qRT-PCR). MCP-1 and MIP-2 relative mRNA expression levels were elevated in the I/R group compared to those in the Sham group (see Fig. 2g,h). However, VNS treatment lowered the relative mRNA expression levels of MCP-1 and MIP-2 compared with those in the I/R group (see Fig. 2g,h). These data suggest that VNS significantly inhibited inflammation in the serum and kidneys after hepatic I/R injury.

### VNS significantly alleviated oxidative stress in the kidneys after hepatic I/R injury

**Figure 3:**
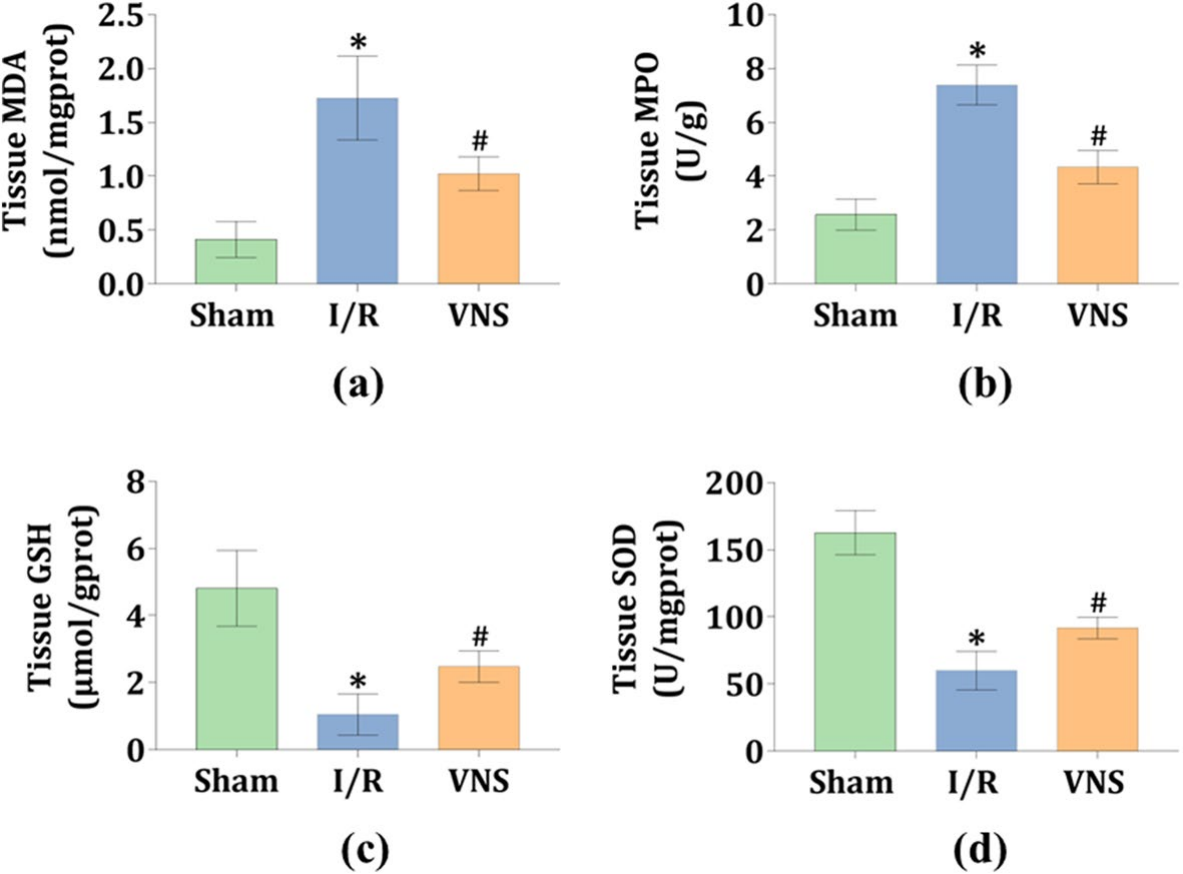
VNS attenuates oxidative stress in the kidneys after hepatic I/R injury. The effect of VNS on the levels of (**a**) MDA and (**c**) GSH in kidney tissues is shown. The effect of VNS on the activity of (**b**) MPO and (**d**) SOD in kidney tissues is shown. Data are expressed as mean ± SD. *p < 0.05 versus the Sham group; ^#^p < 0.05 versus the I/R group. *MDA* myeloperoxidase, *MPO* malondialdehyde, *GSH* glutathione, *SOD* superoxide dismutase.

Oxidative stress leads to overproduction of reactive oxygen species (ROS), formation of myeloperoxidase (MDA) and malondialdehyde (MPO), and decreased expression of anti-oxidative enzymes, such as glutathione (GSH) and superoxide dismutase (SOD). The levels of MDA and GSH, as well as the activity of MPO and SOD, were examined to evaluate oxidative stress in the kidneys. The level of MDA and the activity of MPO were significantly increased in the I/R group compared to the Sham group (see Fig. 3a,b). In contrast, when compared to the I/R group, VNS treatment greatly reversed these changes during the hepatic I/R process (see Fig. 3a,b). Compared with the Sham group, the level of GSH and the activity of SOD were noticeably decreased in the I/R group (see Fig. 3c,d). However, this decrease was significantly reversed by VNS treatment during the hepatic I/R process when compared to the I/R group (see Fig. 3c,d). These data suggest that VNS significantly alleviated oxidative stress in the kidneys after hepatic I/R injury.

### VNS significantly improved redox status in the kidneys after hepatic I/R injury

**Figure 4:**
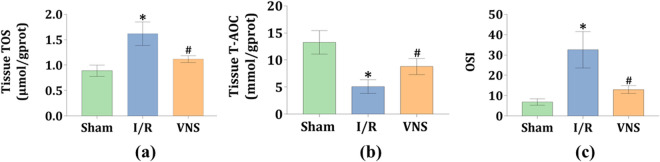
VNS improves redox status in the kidneys after hepatic I/R injury. The effect of VNS on the levels of (**a**) TOS and (**b**) T-AOC in kidney tissues is shown. (**c**) The OSI values is shown. Data are expressed as mean ± SD. *p < 0.05 versus the Sham group; ^#^p < 0.05 versus the I/R group. *TOS* total oxidant status, *T-AOC* total antioxidant capacity, *OSI* oxidative stress index.

To further assess the redox status, the levels of total oxidant status (TOS) and total antioxidant capacity (T-AOC) in the kidneys were determined. The level of TOS was significantly higher, and the level of T-AOS was markedly lower in the I/R group than in the Sham group (see Fig. 4a,b). However, when compared to the I/R group, these changes were greatly reversed by VNS treatment during the hepatic I/R process (see Fig. 4a,b). In parallel, higher oxidative stress index (OSI) values were observed in the I/R group than in the Sham group (see Fig. 4c). However, lower OSI values were observed in the VNS group than in the I/R group (see Fig. 4c). These data indicate that VNS prominently improved the redox status in the kidneys after hepatic I/R injury.

### VNS significantly reduced apoptosis in the kidneys after hepatic I/R injury

**Figure 5:**
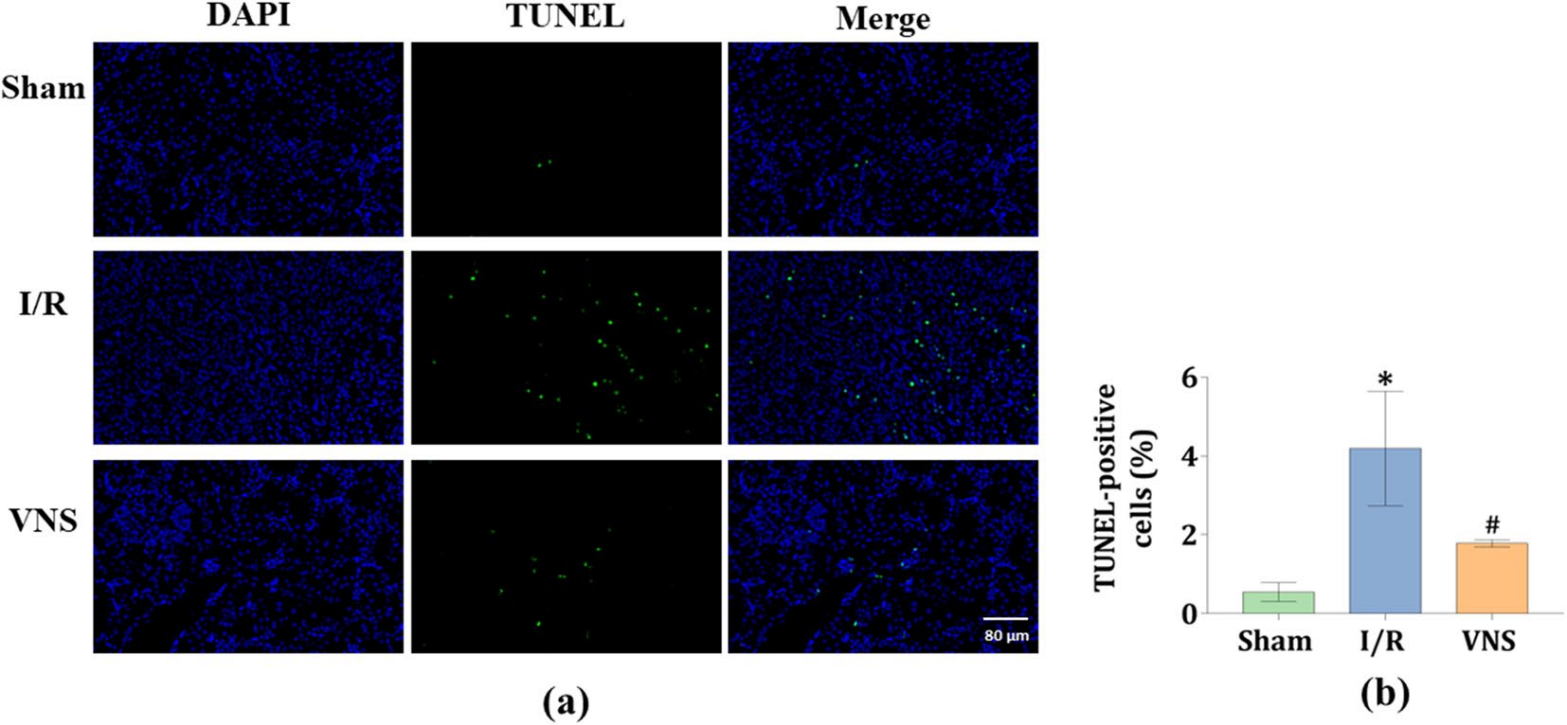
VNS reduces cell apoptosis in kidneys after hepatic I/R injury. (**a**) Representative micrographs of immunofluorescence staining for DAPI (blue) and TUNEL (green) in kidney cell nuclei from the three groups are shown. (**b**) Quantification of kidney cell apoptosis from the three groups is shown. *p < 0.05 vs. the Sham group; ^#^p < 0.05 vs. the I/R group. Fluorescence microscope (× 400 magnification).

To detect apoptotic cells in the kidneys among the three groups, the terminal deoxynucleotidyl transferase-mediated dUTP nick end labeling (TUNEL) staining was performed. The representative micrographs of immunofluorescence staining for DAPI (blue) and TUNEL (green) in kidney cell nuclei from the three groups are shown in Fig. 5a. Compared with the Sham group, the percentage of TUNEL-positive cells was markedly increased in the I/R group (see Fig. 5b). However, when compared to the I/R group, the VNS group showed an obvious reduction in the percentage of TUNEL-positive cells (see Fig. 5b).

**Figure 6:**
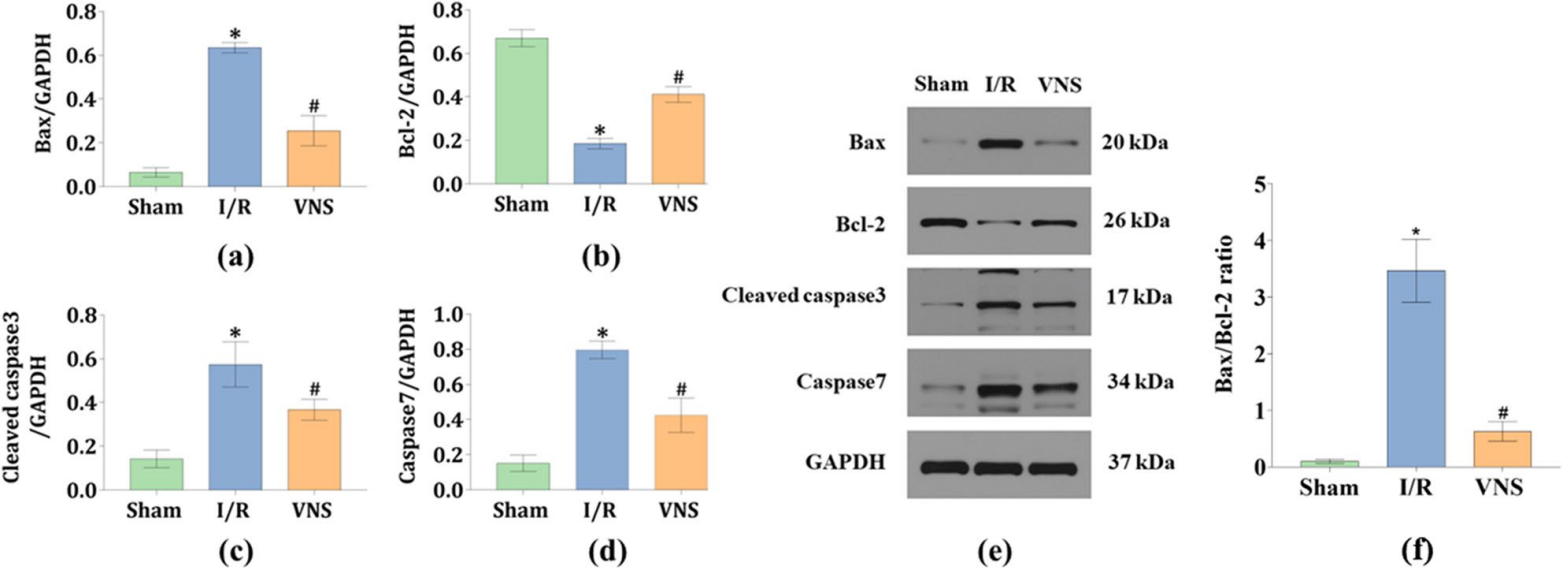
VNS reduces cell apoptosis by regulating Bax, Bcl-2, Cleaved caspase3, caspase7, and Bax/Bcl-2 ratio. (**e**) Representative blots and relative protein levels of (**a**) Bax, (**b**) Bcl-2, (**c**) Cleaved caspase3, and (**d**) caspase7 in kidney tissues from the three groups are shown. (**f**) Bax/Bcl-2 ratio is shown. *p < 0.05 vs. the Sham group; ^#^p < 0.05 vs. the I/R group. Some blots were cut before hybridization with antibodies to optimize the use of the samples.

Western blotting was performed to further evaluate apoptosis in the kidneys. Compared with the Sham group, the relative protein expression level of Bax, which induces apoptosis, was significantly increased in the I/R group and the relative protein expression level of Bcl-2, which inhibits apoptosis, was markedly decreased in the I/R group (see Fig. 6a,b). The relative protein expression levels of cleaved caspase3 and caspase7 were greatly increased in the I/R group (see Fig. 6c,d). Meanwhile, it has been demonstrated that the Bax/Bcl-2 ratio may be more important than either protein alone in determining apoptosis^15,16^. The Bax/Bcl-2 ratio was greatly increased in the I/R group (see Fig. 6f). VNS reversed these changes as described above, when compared to the I/R group (see Fig. 6a–d,f). The representative blots and relative protein levels of Bax, Bcl-2, Cleaved caspase3, and caspase7 in kidney tissues from the three groups are shown in Fig. 6e. These data suggest that VNS significantly reduced apoptosis in the kidneys after hepatic I/R injury.

### VNS significantly enhanced the Nrf2/HO-1 signaling pathway in the kidneys after hepatic I/R injury

**Figure 7:**
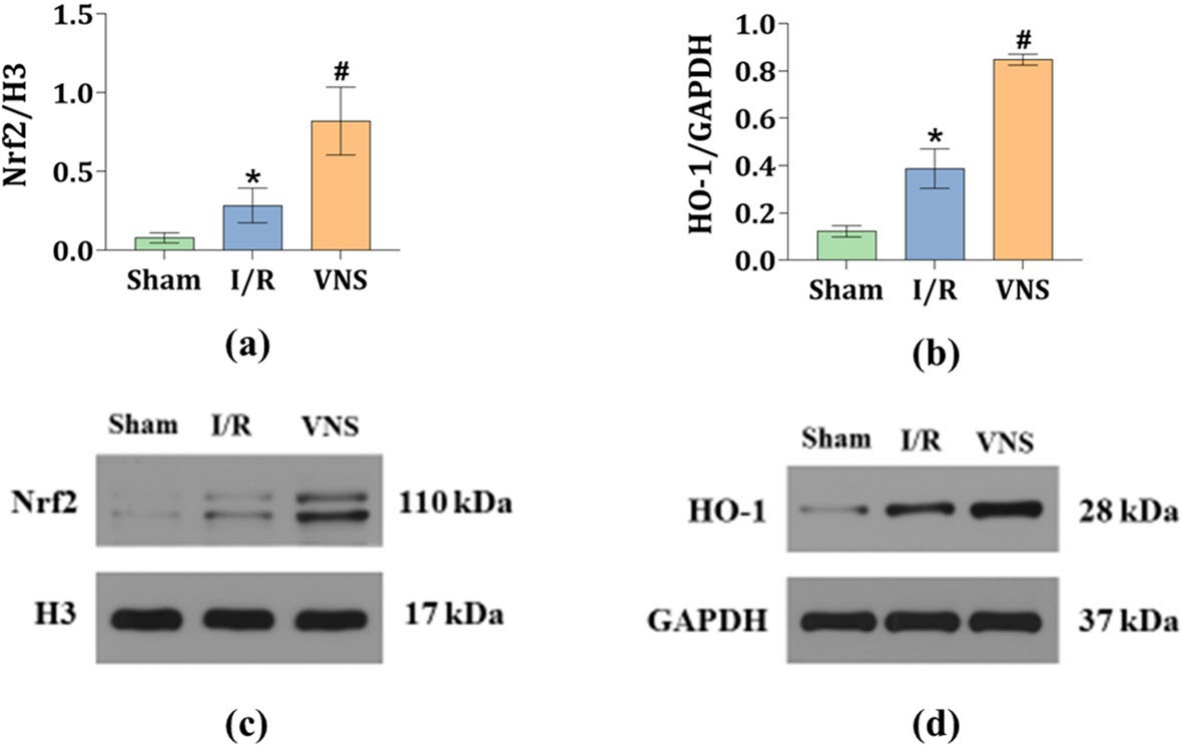
VNS activates the Nrf2/HO-1 signaling pathway in the kidneys after hepatic I/R injury. (**c**, **d**) Representative blots and relative protein levels of (**a**) Nrf2 and (**b**) HO-1 in kidney tissues from the three groups are shown. *p < 0.05 vs. the Sham group; ^#^p < 0.05 vs. the I/R group. *Nrf2* nuclear factor erythroid 2-related factor 2, *HO-1* heme oxygenase-1. Some blots were cut before hybridization with antibodies to optimize the use of the samples.

Western blotting was used to analyze protein expression levels in the three groups. Compared with the Sham group, hepatic I/R injury markedly increased the relative protein expression levels of nuclear factor erythroid 2-related factor 2 (Nrf2) and hemeoxygenase-1 (HO-1) in the kidneys (see Fig. 7a–d). In contrast, when compared to the I/R group, VNS greatly augmented the increases described above (see Fig. 7a–d). These data suggest that the Nrf2/HO-1 signaling pathway was activated in the kidneys after hepatic I/R injury and greatly enhanced by VNS treatment during the hepatic I/R process.

## Discussion

The essential findings of the present study are that hepatic I/R injury can induce remote kidney injury and that VNS treatment protects against AKI after hepatic I/R injury. Notably, we demonstrated for the first time that VNS significantly attenuated inflammation, oxidative stress, and apoptosis in the kidneys during the hepatic I/R process. Additionally, VNS was firstly noted to facilitate the activation of the Nrf2/HO-1 signaling pathway in the kidneys after hepatic I/R injury.

AKI, also regarded as acute renal failure, is characterized by a sudden reduction in renal function or glomerular filtration rate, evolving from early injury to severe damage^6^. It can result in chronic kidney disease or even kidney failure, requiring kidney replacement therapy. The kidneys are vulnerable to a series of challenges from remote hepatic I/R injury, owing to their abundant blood perfusion. Animal and clinical evidence of causality between hepatic I/R injury and AKI has been well documented. In rodents, hepatic I/R injury has been proven to cause AKI^17^. In humans, a large single-center, case-controlled study has shown the same trend in increasing mortality and morbidity of AKI during the LT perioperative period^5^. Therefore, it is meaningful to progress an effective approach to arrest the progression of AKI induced by hepatic I/R injury.

It has been demonstrated that the pathogenesis of AKI was associated with the heightened activity of the sympathetic nervous system^18^. This was also confirmed in a previous study where in the initial 24 h-period following hepatic I/R injury, the occurrence of AKI was attributed to intrarenal vasoconstriction resulting from splanchnic vasodilatation following portal hypertension^19^. These observations indicated that activation of the sympathetic nervous system negatively influences kidney function. Therefore, the activation of the vagus nerve system, which naturally offsets sympathetic vasoconstrictive activity, is an optional approach to restore autonomic regulatory function, result in anti-inflammatory effects, and modulate centrally mediated mechanisms. The evidence from Inoue et al. also suggested that a therapeutic VNS approach may prevent AKI^11^. Hence, we originally proposed the hypothesis that VNS treatment might protect against AKI after hepatic I/R injury. Meanwhile, it has already been revealed that mice subjected to severe hepatic I/R injury develop AKI characterized by immediate kidney peritubular capillary endothelial cell apoptosis with subsequent kidney proximal tubular necrosis and inflammation^19^. Whether VNS could block these pathways to exert protective effects against AKI induced by hepatic I/R injury remains unknown.

In the development of hepatic I/R injury-induced AKI, a substantial systematic inflammatory response always bears the brunt. Acute liver injury following hepatic I/R injury can cause a systemic inflammatory response, resulting in remote kidney injury^20^. This finding is supported by the results of Park et al. They observed that hepatic I/R injury was attributed to a significant upregulation of pro-inflammatory cytokines in the kidneys^21^. The anti-inflammatory function of the vagus nerve is mediated through several pathways, some of them still debated^22^. Of these, the cholinergic anti-inflammatory pathway (CAP) has maybe been studied most extensively. In recent years, VNS at low frequency has been used to activate the CAP to produce anti-inflammatory effects in kidney by inhibiting the production of pro-inflammatory cytokines^23,24^. In our study, we chose the left cervical vagal trunk (efferent fibres) as the stimulating target, which can release acetylcholine to activate the CAP. Our data showed that compared with I/R group, VNS treatment greatly lowered the levels of TNF-α, IL-6, and IL-1β in serum and kidneys. Meanwhile, the relative mRNA expression levels of MCP-1 and MIP-2 also decreased. Therefore, we hypothesized that VNS may ameliorate inflammation in the serum and kidneys after hepatic I/R injury.

Oxidative stress is another crucial contributor to the pathogenesis of hepatic I/R injury-induced AKI. During I/R, ROS are massively released and various ROS subsequently initiate lipid peroxidation reactions, contributing to inflammation activation and tissue damage. A previous study demonstrated that hepatic I/R injury can acutely induce a remote renal cortical oxidative stress response^25^. Meanwhile, MDA, serves as an oxidative stress marker, has been proven to increase in kidneys after hepatic I/R injury^18^. Accumulating evidence from past studies suggested that VNS can reduce oxidative stress in multiple organs, such as kidney, liver, and skeletal muscle^9,26^. Based on the above findings, we attempted to explore whether VNS could alleviate kidney oxidative stress caused by hepatic I/R injury. In this study, our data showed that compared with the I/R group, VNS increased GSH level and SOD activity, and decreased MDA level and MPO activity in the kidneys after hepatic I/R injury. Meanwhile, compared with the I/R group, VNS decreased TOS level, increased T-AOC level, and decreased OSI value. Therefore, we hypothesized that VNS may protect against hepatic I/R injury-induced AKI, partly through its antioxidative properties.

Kidney apoptosis is a hallmark of AKI^27^. It has been demonstrated that hepatic I/R injury can cause kidney peritubular capillary endothelial cell apoptosis^19^. Lai et al. revealed that VNS exerted protective effects on acute liver injury after kidney I/R injury, probably by suppressing cellular apoptosis^14^. This evidence indicated that VNS can inhibits apoptosis and plays an important role in remote organ injury induced by I/R injury. Kidney is another important organ of the liver-kidney axis, we thus speculated that VNS could suppress kidney apoptosis and protect against AKI induced by hepatic I/R injury. Our data showed that compared with the I/R group, the protein expression levels of Bax, cleaved caspase3 and caspase7 decreased, the Bax/Bcl-2 ratio decreased, the protein expression level of Bcl-2 increased. Therefore, we hypothesized that VNS may alleviate apoptosis in the kidneys after hepatic I/R injury.

Crosstalk between organs affects each other’s functioning via various pathways, including endocrine, neural, and direct cell–cell signaling pathways^28^. Nrf2, a transcription factor, mediates cellular defense against oxidative stress in organs. Under physiological cellular conditions, Nrf2 is sequestered in the cytoplasm in association with its protein inhibitor, Kelth-like ECH-associated protein-1. Under pathological cellular conditions, Nrf2 is transferred into the nucleus where it upregulates relevant cytoprotective enzymes, such as HO-1^29^. Kudoh et al. reported that in a rat model of hepatic I/R injury, the depletion of Nrf2 aggravated inflammation and lead to oxidative stress and apoptosis^30^. It is widely accepted that Nrf2 activation induces HO-1 transcription, which has been demonstrated to be closely involved in alleviating AKI by minimizing cellular oxidative stress in a recent finding^31^. Meanwhile, a previous research verified that VNS-mediated attenuation of AKI depends on α7 nicotinic acetylcholine receptor (α7nAChR)-positive splenocytes^11^. As the target of the CAP, α7nAChR activation can directly induce the Nrf2/HO-1 signaling pathway^32,33^. As noted above, the Nrf2/HO-1 signaling pathway may be an important signaling pathway for the protection of VNS in hepatic I/R injury-induced AKI. Our data showed that VNS strongly enhanced the protein expression levels of Nrf2 and HO-1 in the kidneys. It is possible to conclude that the Nrf2/HO-1 signaling pathway may serve as an important mediator of the protective effects of VNS in the setting of AKI following hepatic I/R injury.

The data from our study showed that VNS may be a potential clinical treatment for hepatic I/R injury-induced AKI. In recent years, it has been demonstrated that VNS can protect against hepatic I/R injury^10,34^. Undoubtedly, the effective treatment of primary disease can certainly provide a benefit in the treatment of the secondary injury. However, it is also noteworthy that VNS has a direct therapeutic effect on hepatic I/R injury-induced AKI. Traditional VNS is always invasive and requires device implantation; therefore, the application of VNS is widely restricted. Auricular VNS, a non-invasive VNS, has been shown to obtain the similar effects as invasive VNS^35,36^. Considering these observations together, non-invasive VNS might be a prospective clinical treatment for hepatic I/R injury-induced AKI.

There are limitations to our study which ought to be mentioned. First, our study is only a preliminary exploration of the mechanisms underlying the VNS protective effect on hepatic I/R injury-induced AKI. We failed to determine the causality of VNS directly on AKI and clearly report the precise mechanisms. Second, we chose pentobarbital to anesthetize all experimental animals, which may affect the autonomic nervous system function. Third, different VNS frequencies, intensities, and durations have been shown to exert different therapeutic effects. However, only one stimulation parameter was used in the present study. It is unclear if there is an enhanced effect with a different parameter. Finally, this experiment was tested in only one animal model, and extrapolation from rats to humans can be difficult.

**Figure 8:**
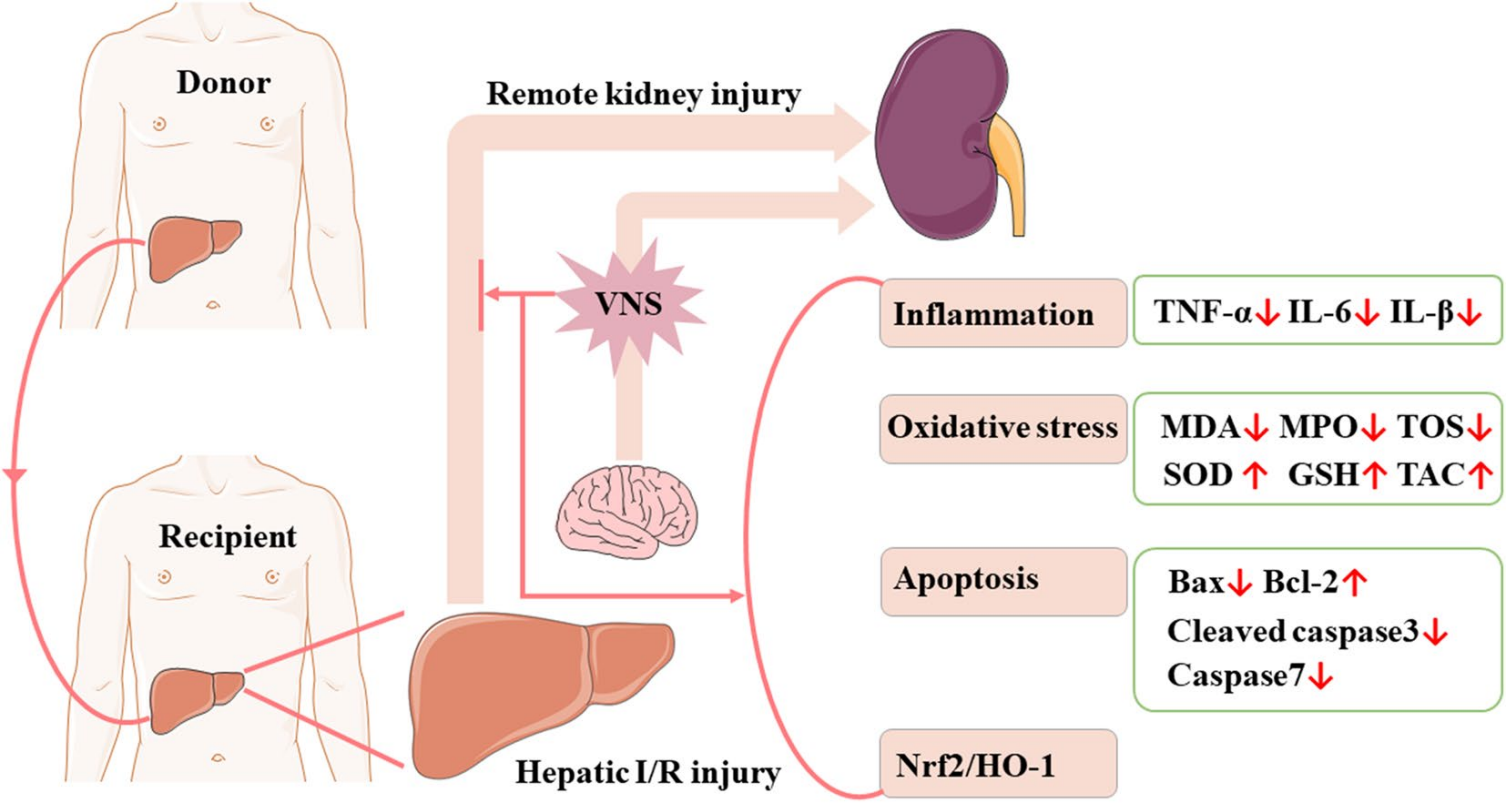
Schematic diagram depicting the protective effect of VNS on AKI after hepatic I/R injury and its potential mechanisms. VNS protects against AKI after hepatic I/R injury by suppressing inflammation, oxidative stress, and apoptosis probably via the Nrf2/HO-1 signaling pathway.

In conclusion, our data suggested that VNS can protect against AKI after hepatic I/R injury. The potential mechanisms may involve inhibiting inflammation, suppressing oxidative stress, and reducing apoptosis. Additionally, VNS greatly activated the Nrf2/HO-1 signaling pathway in the kidneys. Taken together, VNS may attenuate hepatic I/R injury-induced AKI by suppressing inflammation, oxidative stress, and apoptosis probably via the Nrf2/HO-1 signaling pathway (see Fig. 8). With the development of non-invasive VNS, VNS might provide a novel clinical treatment for patients with hepatic I/R injury-induced AKI.

## Methods

### Animal preparation and experimental groups

**Figure 9:**
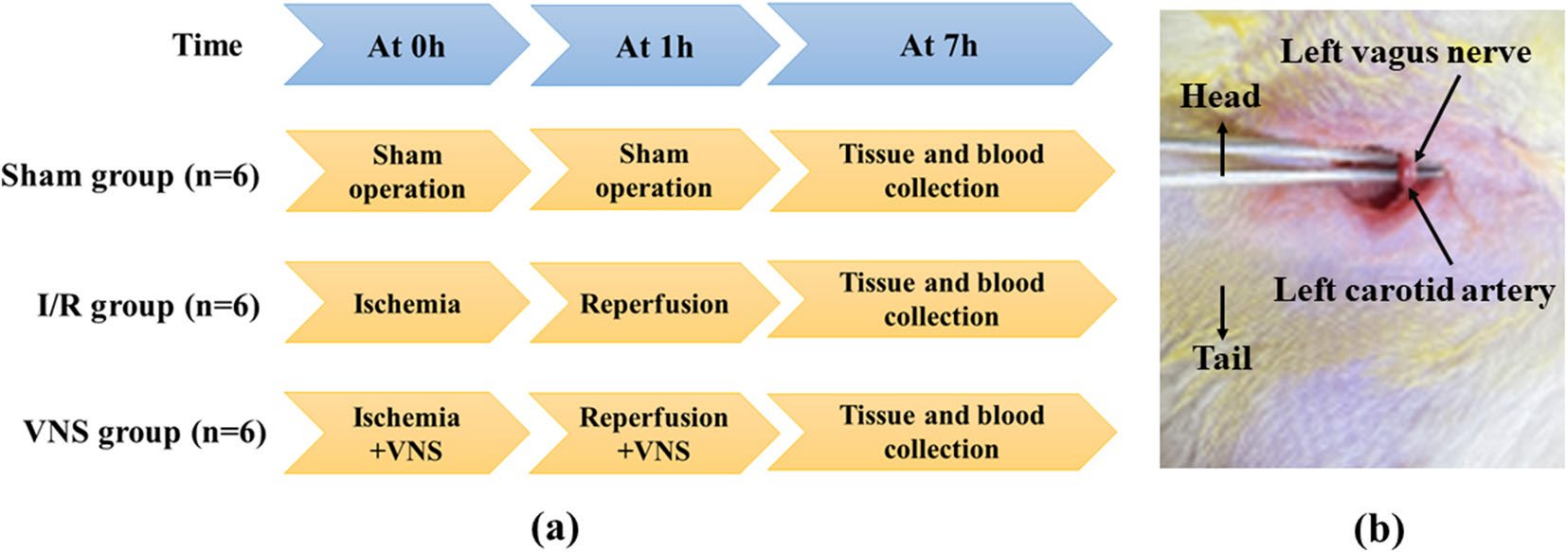
Experimental protocol (**a**) and location of the left vagus nerve (**b**). *Sham* sham operation, *I/R* ischemia–reperfusion, *VNS* vagus nerve stimulation.

All animal experiments complied with the ARRIVE guidelines. Healthy male Sprague–Dawley rats (each weighing 280–350 g) used in this study were conducted in accordance with the Guide for the Care and Use of Laboratory Animals by the US National Institutes of Health (NIH Publication No. 85-23, revised 1996) and approved by the Animal Care and Use Committee of the Second Xiangya Hospital of Central South University. Rats were housed in an environment with controlled temperature (22 °C ± 1 °C) and relative humidity (50% ± 5%) on a 12:12-h light/dark cycle, with free access to sterile food and water. Eighteen rats were randomly allocated into three groups and received different treatments: sham group (sham operation, n = 6); I/R group (hepatic I/R with sham VNS, n = 6); and VNS group (hepatic I/R with VNS, n = 6). A flowchart of the experimental design is shown in Fig. 9a, and the location of left vagus nerve is shown in Fig. 9b. During the whole experiment, the body surface electrocardiogram of rats was recorded with a TECHMAN biological signal acquisition system (BL-420F, Chengdu City, China).

### Preparation of hepatic I/R injury model

According to the method of Zhang et al*.*^10^, we established an acute segmental (70%) hepatic I/R injury model in this experiment. All rats fasted overnight before surgery. On the day of the operation, all rats were weighed and anesthetized with 1% pentobarbital sodium (40 mg/kg body weight) by intraperitoneal injection. Briefly, a midline abdominal incision was made, and an atraumatic vascular clamp was used to interrupt the arterial and portal venous blood supply to the left and median liver lobes. The clamp was removed after 1 h to initiate a 6 h hepatic reperfusion period. Throughout the operation, the body temperature was monitored using a rectal probe and maintained at 37 °C with a heating pad. At the end of the experiment, all rats were anesthetized by inhaling methoxyflurane and sacrificed by exsanguination.

### Vagus nerve stimulation

In this study, we chose the left cervical vagal trunk as the stimulating target, which was carefully separated from the surrounding connective tissues and stimulated (SDZ-IIB, Hwato, Suzhou City, China) through use of a bipolar silver wire electrode. Subsequently, continuous low-frequency stimulation (HFS, 20 Hz, 0.2 ms in duration, square waves) were delivered. VNS was performed during the entire hepatic I/R process^12^. The stimulus intensity should achieve a 10% reduction in the sinus rate and adjust hourly^10,37^.

### Collection of blood and tissue samples

At the end of reperfusion, blood samples were drawn from the inferior caval vein and centrifuged to separate the serum (3000 rpm, 15 min, 4 °C). At the end of the experiment, kidney tissues were collected quickly from each rat. The left kidney was coronally dissected. Half of the kidney was fixed with paraformaldehyde and the other half was frozen with liquid nitrogen. Both blood and tissue samples were stored at − 80 °C until the biochemical assays, molecular biological detection, and histological examination.

### Assessment of function and determination of inflammatory cytokines

Kidney function and inflammation after hepatic I/R injury were determined by measuring the serum levels of BUN and creatinine Cr, which were detected using commercially available kits (Nanjing Jiancheng Bioengineering Institute, Nanjing City, China). The serum and kidney tissues levels of TNF-α, IL-6, and IL-1β were analyzed by ELISA (ELK Biotechnology, Wuhan City, China) as per the manufacturer’s instructions.

### Measurements of oxidative stress in kidney tissues after hepatic I/R injury

The levels of MDA, MPO, GSH, and SOD were measured in the kidney tissues using different assay kits (Nanjing Jiancheng Bioengineering Institute, Nanjing City, China). To assess the cumulative antioxidant action and the oxidative stress level, the levels of TOS and T-AOC were determined in the kidney tissues using commercially available kits (Nanjing Jiancheng Bioengineering Institute, Nanjing City, China). The OSI, represented by the ratio of TOS to T-AOC, is an indicator of the degree of oxidative stress. The OSI value is calculated according to the formula: OSI = TOS (μmol/gprot)/T-AOC (mmol/gprot) × 100.

### Histological examination and quantification of kidney injury after hepatic I/R injury

Kidney tissue samples from different groups were separated and fixed with 4% paraformaldehyde for 24 h. After automated dehydration through a graded alcohol series, the kidney slices were embedded in paraffin. Sections (4-μm thick) were stained with hematoxylin and eosin (H&E) to determine morphological changes in the kidney tissues. Finally, images of the sections were captured under a light microscope (× 400 magnification). Histological scoring was performed by grading tubular necrosis, loss of brush border, cast formation, and tubular dilatation as follows: 0, none; 1, < 10%; 2, 11–25%; 3, 26–45%; 4, 46–75%; and 5, > 76%^38^. Ten views from each tissue sample among the three groups were randomly screened, and the mean was regarded as the representative value of the sample (see Supplementary Information). TUNEL staining was performed to determine apoptosis. Kidney tissue sections (4-μm thick) were deparaffinized in xylene and then hydrated in graded ethanol. After treatment with protease K, the sections were rinsed with phosphate-buffered saline and then incubated completely in a TUNEL reaction reagent for 60 min at 37 °C. After washing three times with phosphate-buffered saline, the sections were treated with 4,6-diamidino-2-phe-nylindole and incubated in the dark. The stained sections were analyzed using a fluorescence microscope (Nikon DS-U3, Tokyo, Japan). Cells with nuclei containing irregular green particles were regarded as TUNEL-positive cells, and the percentage of TUNEL-positive cells was recorded and averaged in three random high-power fields (× 400 magnification) (see Supplementary Information).

### Western blotting analysis

The protein expression levels of Bax, Bcl-2, cleaved caspase 3, and caspase 7 in kidney tissues, which are often regarded as indicators of apoptosis, were assessed to determine the effect of VNS on kidney apoptosis in the hepatic I/R injury model. The level of HO-1 was measured in kidney lysates, and Nrf2 levels were detected in nuclear lysates. Briefly, cells and tissues were completely homogenized in a lysis buffer. Total proteins were extracted from the supernatant and extracted into 1.5 ml EP tubes. Equal amounts of denatured protein solution were separated by sodium dodecyl sulfate polyacrylamide gel electrophoresis and subsequently transferred to polyvinylidene fluoride membranes. The membranes were blocked with 5% nonfat milk for 1 h at 25 °C. Some blots were cut before hybridization with antibodies to optimize the use of the samples. And then incubated with rabbit anti-glyceraldehyde-3-phosphate dehydrogenase antibody (GAPDH, 1:10,000 dilution, Abcam, Cambridge, UK), rabbit anti-Histone H3 antibody (1:3000 dilution, CST, Boston, USA), rabbit anti-Bax antibody (1:2000 dilution, CST, Boston, USA), rabbit anti-Bcl-2 antibody (1:2000 dilution, Abcam, Cambridge, UK), rabbit anti-cleaved caspase3 antibody (1:500 dilution, Affbiotech, Shanghai City, China), rabbit anti-caspase7 antibody (1:500 dilution, Abcam, Cambridge, UK), rabbit anti-nuclear factor erythroid 2-related factor 2 antibody (Nrf2, 1:500 dilution, Abcam, Cambridge, UK), and rabbit anti-heme oxygenase-1 antibody (HO-1, 1:2000 dilution, CST, Boston, USA) overnight at 4 °C. After washing in Tris-buffered saline containing Tween, the polyvinylidene fluoride membranes were incubated with horseradish peroxidase-goat anti-rabbit secondary antibodies at room temperature for 1 h. Finally, CAPDH and Histone H3 act as loading controls^39^, the relative protein expression was standardized to GAPDH and Histone H3 from the same sample and quantified using a AlphaEase FC image analyzer software (Alpha Innotech, California, USA).

### Quantitative real-time polymerase chain reaction (qRT-PCR)

The mRNA levels of MCP-1 and MIP-2 were evaluated by qRT-PCR. Total RNA was extracted from kidney tissues using TRIpure Total RNA Extraction Reagent (ELK Biotechnology, Wuhan City, China) following the manufacturer’s protocol. First-strand cDNA was synthesized using the EntiLink™ 1st Strand cDNA Synthesis Kit (ELK Biotechnology, Wuhan City, China). Target gene expression levels were measured with EnTurbo™ SYBR Green PCR SuperMix (ELK Biotechnology, Wuhan City, China) using a StepOne™ RT-PCR thermocycler (Life Technologies, Massachusetts, USA). The mRNA levels of MCP-1 and MIP-2 were normalized to the β-actin mRNA level in the same sample and calculated using the Delta-Delta-CT method. The primer sequences were as follows: MCP-1, forward: 5′-GGCCTGTTGTTCACAGTTGCT-3′; reverse, 5′-GCCGACTCATTGGGATCATC-3′; MIP-2, forward: 5′-GTCAATGCCTGACGACCCTAC-3′, reverse: 5′-CCTTCCCAGGTCAGTTAGCCT-3′; and β-actin, forward: 5′- CGTTGACATCCGTAAAGACCTC-3′, reverse: 5′-TAGGAGCCAGGGCAGTAATCT-3′.

### Statistical analysis

All continuous variables were presented as mean ± standard deviation (SD). GraphPad Prism version 7.0 software (GraphPad Software, Inc. San Diego, CA) was used for statistical analysis and graphing. Between-group differences were analyzed by one-way analysis of variance and two-tailed p ≤ 0.05, indicating statistical significance (Supplementary figures).

## Supplementary Information

Supplementary Figures.

## Data Availability

The raw data supporting the conclusions of this article will be made available by the authors, without undue reservation.

## Abbreviations

I/R: Ischemia/reperfusion
LT: Liver transplantation
AKI: Acute kidney injury
VNS: Vagus nerve stimulation
BUN: Blood urea nitrogen
Cr: Creatinine
TNF-α: Tumor necrosis factor alpha
IL-6: Interleukin 6
IL-1β: Interleukin1-beta
MCP-1: Monocyte chemoattractive protein 1
MIP-2: Macrophage inflammatory protein 2
qRT-PCR: Quantitative real-time polymerase chain reaction
ROS: Reactive oxygen species
MDA: Myeloperoxidase
MPO: Malondialdehyde
GSH: Glutathione
SOD: Superoxide dismutase
TOS: Total oxidant status
T-AOC: Total antioxidant capacity
OSI: Oxidative stress index
TUNEL: Terminal deoxynucleotidyl transferase-mediated dUTP nick end labeling
Nrf2: Nuclear factor erythroid 2-related factor 2
HO-1: Hemeoxygenase-1
CAP: Cholinergic anti-inflammatory pathway
α7nAChR: α7 Nicotinic acetylcholine receptor
H&E: Hematoxylin and eosin
SD: Standard deviation
