# Supplementary material for: Vagus nerve stimulation attenuates acute kidney injury induced by hepatic ischemia/reperfusion injury in rats
Source: Sci Rep. 2022 Dec 15;12:21662. doi: 10.1038/s41598-022-26231-w (PMC9755310; doi:10.1038/s41598-022-26231-w)

Supplementary Fig. S1. Light micrographs of kidney tissues. We randomly screened ten views from each tissue sample among the Sham, I/R, and VNS groups. The mean histological score was regarded as the representative value of the sample.

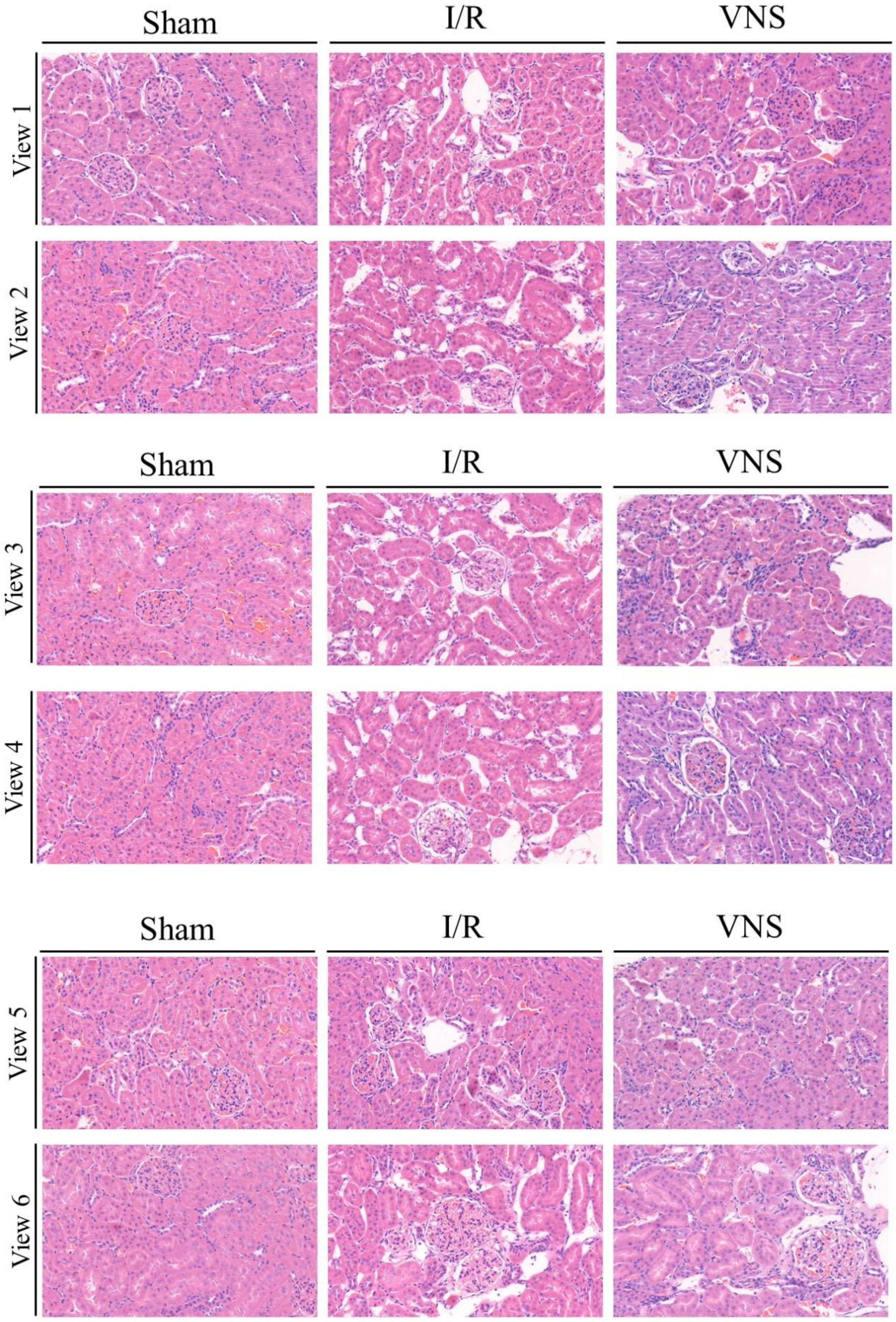

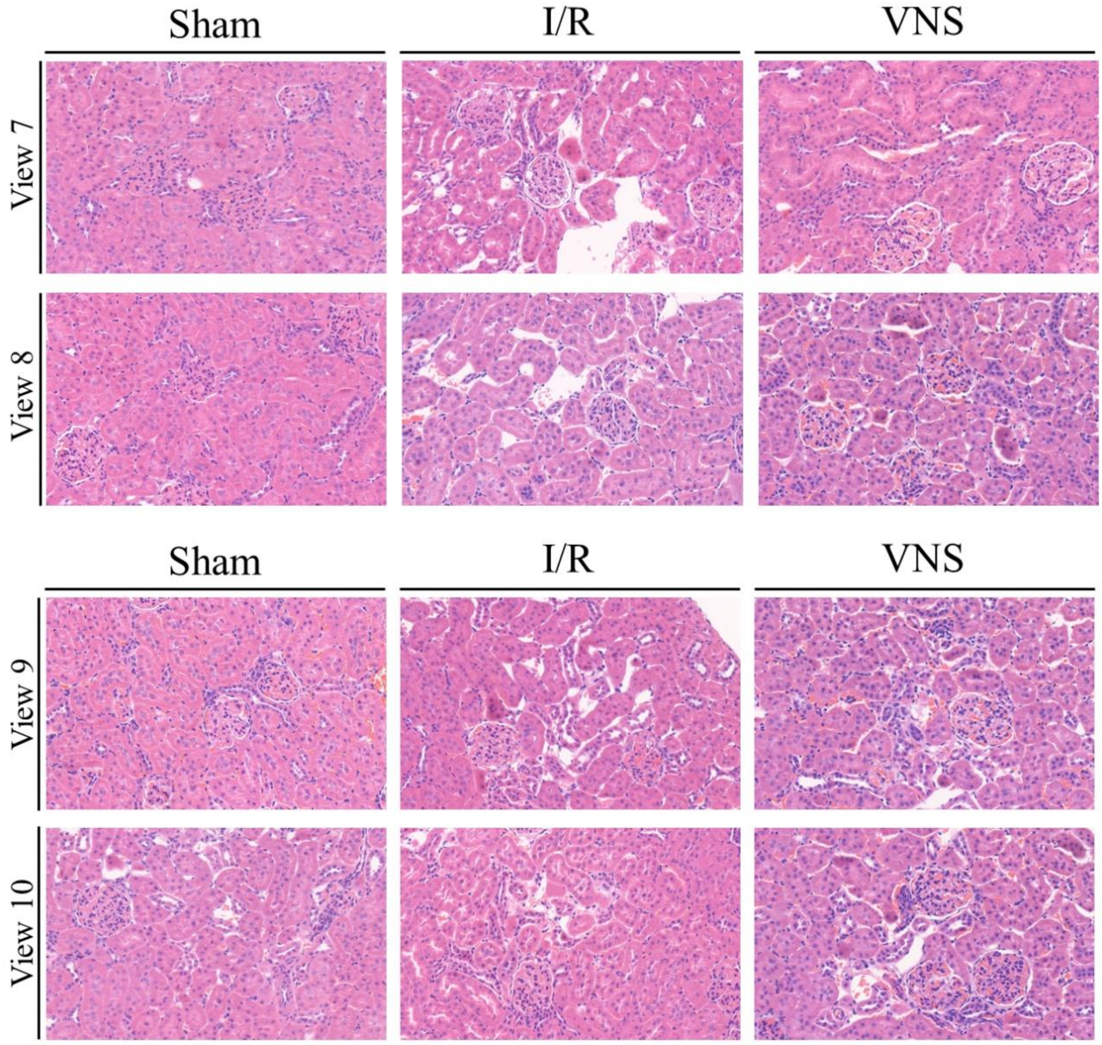
Sham
I/R
VNS
View 7
View 8
Sham
I/R
VNS
View 9
View 10

Supplementary Fig. S2. Fluorescence micrographs of kidney tissues. We randomly screened three views from each tissue sample among the Sham, I/R, and VNS groups. The mean percentage of TUNEL-positive cells was regarded as the representative value of the sample.

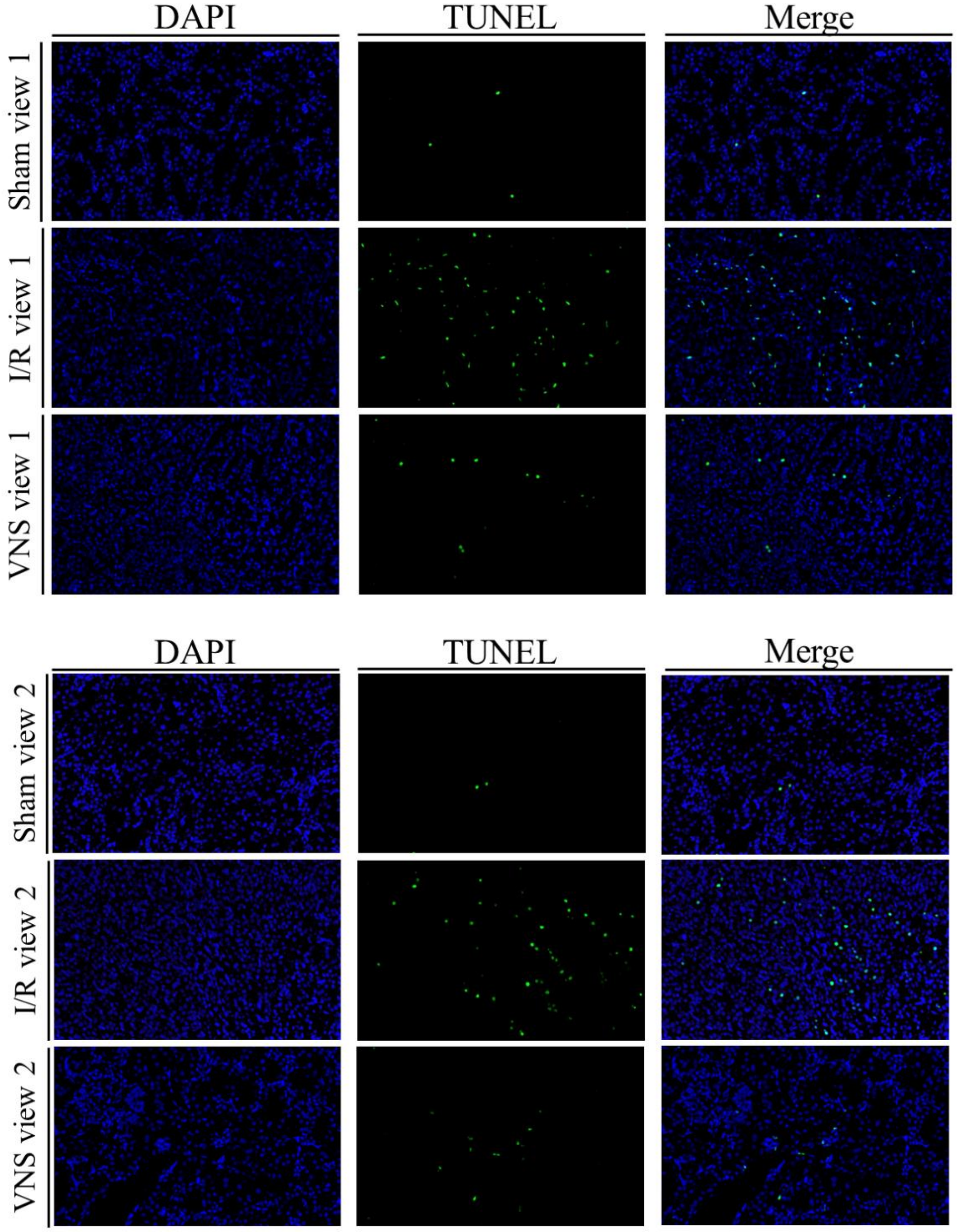

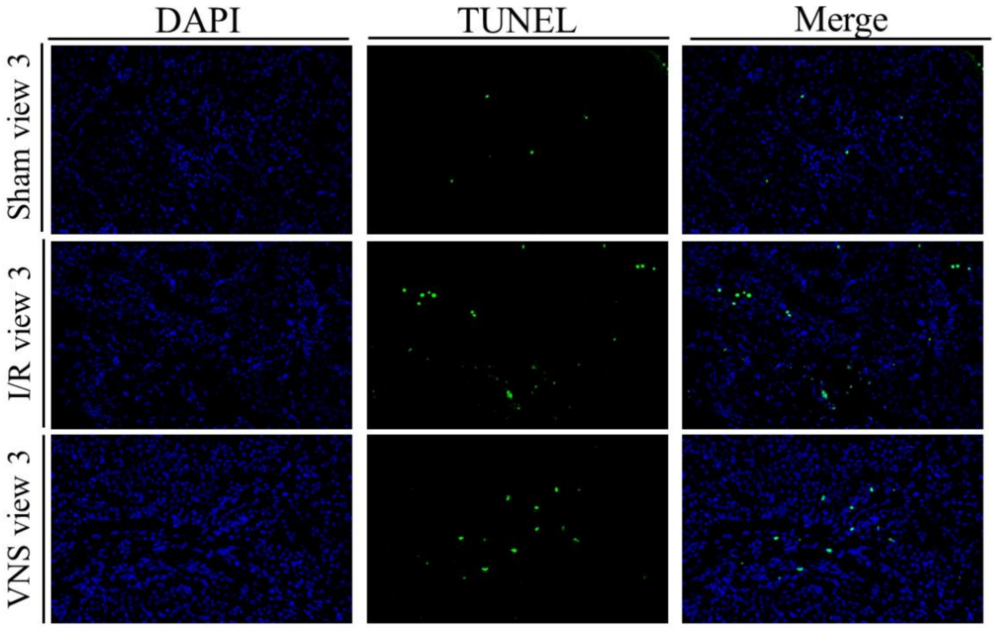
DAPI
TUNEL
Merge
Sham view 3
I/R view 3
VNS view 3

Supplementary Fig. S3. The raw data of Figure (1-7). (a-g) corresponds to Figure (1-7)

(a)

(a) Histological Scoring

Sham	I/R	VNS
1	2	1
1	3	1
0	4	2
1	4	2
1	4	1
0	3	2
1	3	3
0	2	2
1	4	1
0	3	2

(b) Serum BUN (mmol/L)

Sham	I/R	VNS
3.732	13.014	6.603
2.392	13.684	7.943
3.062	12.919	8.134
4.019	15.885	6.507
3.636	13.971	7.464
4.306	12.536	5.742

(c) Serum Cr (μmol/L)

Sham	I/R	VNS
27.753	151.618	94.554
28.629	167.588	124.352
40.607	152.397	108.382
18.502	136.427	102.442
48.592	146.457	94.652
29.895	182.584	89.685

(b)

(a) Serum TNF-α (pg/mL)

Sham	I/R	VNS
66.185	204.858	90.997
41.300	154.540	115.484
39.910	142.712	147.206
21.505	100.362	48.650
47.279	142.303	132.870
67.514	167.536	125.861

(b) Kidney TNF-α (pg/mg pro)

Sham	I/R	VNS
122.719	301.193	218.753
80.699	334.111	222.109
94.401	265.528	219.027
140.129	234.442	151.225
116.502	312.106	196.913
119.531	297.896	147.159

(c) Serum IL-6 (pg/mL)

Sham	I/R	VNS
33.404	149.113	56.593
50.824	153.392	72.990
35.882	100.655	92.920
69.915	90.133	79.726
32.789	148.809	116.462
56.369	97.021	61.343

(d) Kidney IL-6 (pg/mg pro)

Sham	I/R	VNS
161.654	522.613	297.782
91.610	597.503	394.323
144.127	370.577	368.052
149.616	329.540	289.829
221.038	450.212	289.994
209.563	530.787	221.033

(e) Serum IL-1β (pg/mL)

Sham	I/R	VNS
34.179	174.839	68.173
20.281	129.145	98.379
54.160	135.204	110.543
43.784	86.174	86.989
62.827	177.680	130.761
23.241	123.084	47.112

(f) Kidney IL-1β (pg/mg pro)

Sham	I/R	VNS
55.617	129.920	82.194
39.493	184.368	72.515
55.803	148.311	93.741
59.623	110.127	82.482
44.599	119.912	105.335
41.693	149.878	74.750

(g) Relative mRNA level of MCP-1

Sham	I/R	VNS
1	3.28	1.91
0.56	2.93	1.94
1.3	3.81	1.63
1.32	2.47	2.34
1.32	3.44	1.88
0.68	3.4	2.34

(h) Relative mRNA level of MIP-2

Sham	I/R	VNS
1	5.36	3.1
1.62	4.68	2.5
0.66	5.66	3.45
1.35	5.7	3.31
0.81	4.79	3.33
1.24	5	2.76

(c)

(a) Tissue MDA (nmol/mgprot)

Sham	I/R	VNS
0.449	1.747	1.066
0.511	1.256	0.873
0.183	1.576	1.145
0.632	1.409	1.168
0.459	2.197	0.786
0.251	2.160	1.110

(b) Tissue MPO (U/g)

Sham	I/R	VNS
2.230	7.805	3.841
3.044	7.097	4.991
3.540	7.858	4.035
2.389	6.549	4.832
2.035	6.637	4.796
2.283	8.372	3.522

(c) Tissue GSH (μmol/gprot)

Sham	I/R	VNS
4.226	0.305	2.387
3.175	1.679	2.830
5.369	1.745	2.154
4.175	0.389	2.967
6.030	1.064	1.770
5.905	1.168	2.809

(d) Tissue SOD (U/mgprot)

Sham	I/R	VNS
150.788	51.474	96.426
185.539	49.085	97.344
174.605	71.128	89.696
167.764	72.355	85.312
139.627	75.244	101.997
158.550	41.403	80.807

(d)

(a) Tissue TOS (μmol/gprot)

Sham	I/R	VNS
0.78	1.44	1.19
0.87	1.66	1.10
0.85	1.41	1.03
1.01	1.57	1.20
1.04	2.05	1.11
0.79	1.58	1.08

(b) Tissue T-AOC (mmol/gprot)

Sham	I/R	VNS
12.95	5.27	8.81
12.51	3.32	8.73
15.57	5.59	6.26
10.22	5.26	10.81
12.33	7.01	8.87
15.98	4.12	9.33

OSI

Sham	I/R	VNS
6.000	27.395	13.532
5.567	49.842	12.647
8.331	25.252	16.451
8.044	29.813	11.106
8.421	29.191	12.550
4.951	33.930	11.551

(e)

(b) TUNEL-positive cells (%)

Sham	I/R	VNS
0.35	5.86	1.88
0.49	3.37	1.69
0.81	3.33	1.78

(f)

(a) Bax/GAPDH

Sham	I/R	VNS
0.038	0.647	0.323
0.042	0.658	0.208
0.093	0.597	0.258
0.072	0.617	0.349
0.078	0.638	0.175
0.070	0.648	0.218

(b) Bcl-2/GAPDH

Sham	I/R	VNS
0.716	0.162	0.434
0.715	0.155	0.401
0.616	0.218	0.425
0.668	0.188	0.395
0.648	0.192	0.458
0.655	0.201	0.353

(c) Cleaved caspase3/GAPDH

Sham	I/R	VNS
0.168	0.686	0.429
0.187	0.637	0.404
0.090	0.435	0.340
0.163	0.669	0.307
0.152	0.508	0.331
0.095	0.508	0.392

(d) Caspase7/GAPDH

Sham	I/R	VNS
0.118	0.839	0.411
0.108	0.831	0.360
0.184	0.765	0.499
0.101	0.746	0.332
0.205	0.746	0.581
0.192	0.852	0.360

(f) Bax/Bcl-2 ratio

Sham	I/R	VNS
0.053	3.996	0.744
0.058	4.246	0.520
0.151	2.736	0.606
0.108	3.274	0.886
0.121	3.321	0.382
0.106	3.223	0.618

(g)

(a) Nrf2/ H3

Sham	I/R	VNS
0.059	0.174	0.507
0.067	0.208	0.715
0.059	0.182	0.687
0.054	0.341	0.975
0.115	0.411	0.996
0.126	0.397	1.046

(b) HO-1/GAPDH

Sham	I/R	VNS
0.169	0.544	0.874
0.116	0.351	0.858
0.110	0.416	0.845
0.119	0.349	0.840
0.120	0.314	0.809
0.101	0.354	0.863

Supplementary Fig. S4. Full-length original western blot of Figure 6: VNS reduces cell apoptosis by regulating Bax, Bcl-2, Cleaved caspase3, caspase7, and Bax/Bcl-2 ratio. Bax, Bcl-2, Cleaved caspase3, and caspase7 protein expression levels were analyzed by western blotting in the Sham, I/R, VNS groups. GAPDH was used as loading control. Some blots were cut before hybridization with antibodies to optimize the use of the samples.

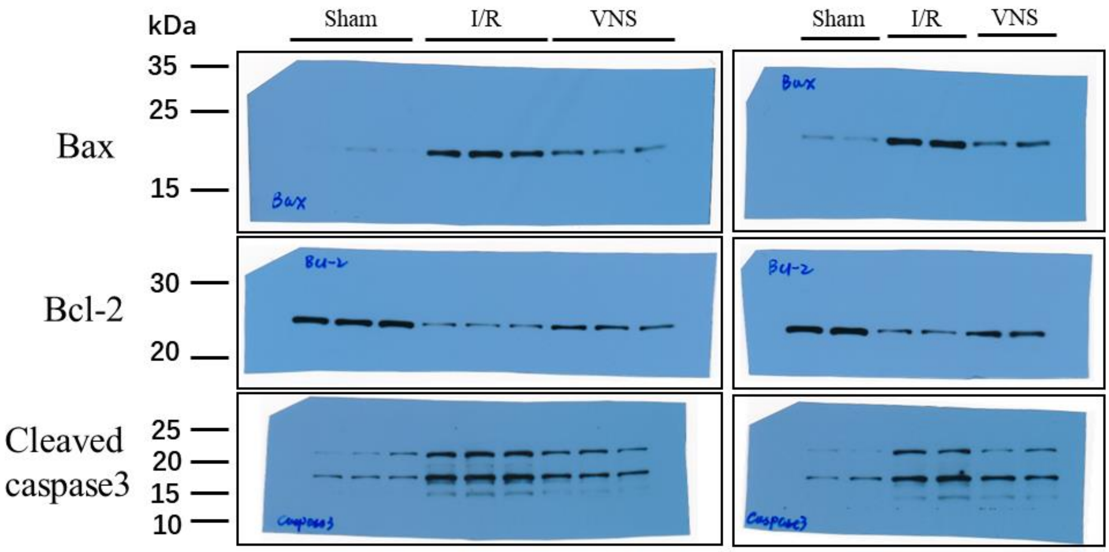

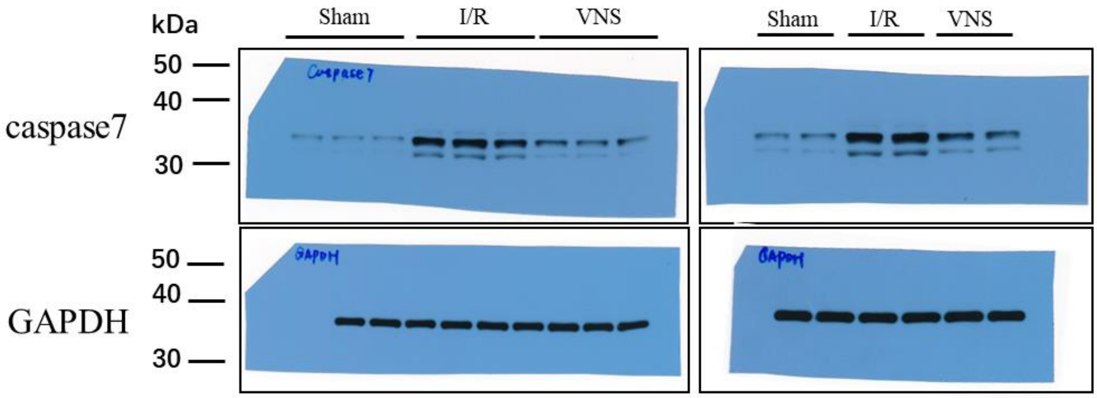

Supplementary Fig. S5. Full-length original western blot of Figure 7: VNS activates the Nrf2/HO-1 signaling pathway in the kidneys after hepatic I/R injury. Nrf2 and HO-1 protein expression levels were analyzed by western blotting in the Sham, I/R, VNS groups. H3 and GAPDH were used as loading control. Some blots were cut before hybridization with antibodies to optimize the use of the samples.

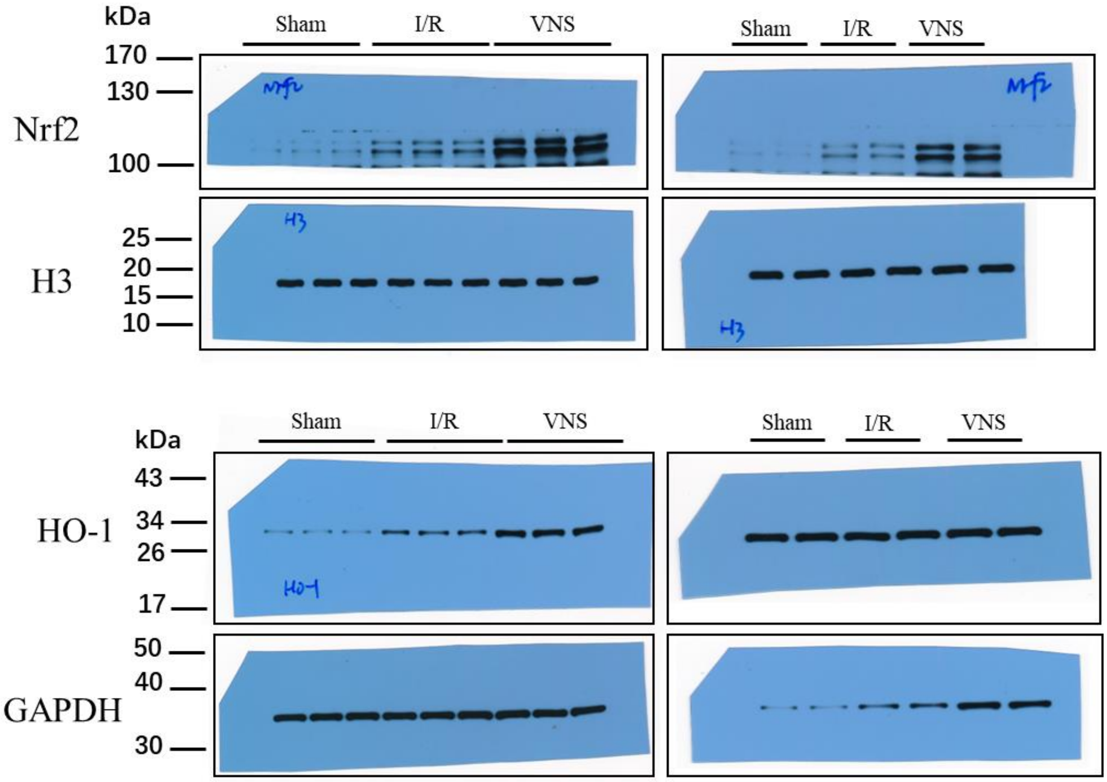

Supplement: Supplementary file 1 — Supplementary Figures. [file 41598_2022_26231_MOESM1_ESM.pdf]
